# Numerical and Experimental Analysis of a Prototypical Thermoelectric Generator Dedicated to Wood-Fired Heating Stove

**DOI:** 10.3390/mi14010145

**Published:** 2023-01-05

**Authors:** Krzysztof Sornek, Karolina Papis-Frączek

**Affiliations:** Department of Sustainable Energy Development, Faculty of Energy and Fuels, AGH University of Science and Technology, Mickiewicza Ave. 30, 30-059 Kraków, Poland

**Keywords:** micro-cogeneration, CHP, wood-fired stove, CFD modeling, thermoelectric generator, TEG

## Abstract

The typical operating range of domestic heating devices includes only heat generation. However, the availability of combined heat and power generation in microscale devices is currently becoming a more and more interesting option. This paper shows the experimental and numerical analysis of the possibility of developing a micro-cogeneration system equipped with a wood-fired heating stove and a prototype of the thermoelectric generator equipped with low-price thermoelectric modules. In the first step, mathematical modeling made it possible to analyze different configurations of the hot side of the thermoelectric generator (computational fluid dynamics was used). Next, experiments have been conducted on the prototypical test rig. The maximum power obtained during the discussed combustion process was 15.9 W_e_ when the flue gas temperature was approximately 623 K. Assuming a case, when such value of generated power occurred during the whole main phase, the energy generated would be at a level of approximately 33.1 Wh_e_, while the heat transferred to the water would be approximately 1 078.0 Wh_th_. In addition to the technical aspects, the economic premises of the proposed solution were analyzed. As was shown, an installation of TEG to the existing stove is economically not viable: the Simply Payback Time will be approximately 28.9–66.1 years depending on the analyzed scenario. On the other hand, the SPBT would be significantly shorter, when the installation of the stove with an integrated thermoelectric generator was considered (approximately 5.4 years). However, it should be noted that the introduction of the power generating system to a heat source can provide fully or partially network-independent operation of the hot water and heating systems.

## 1. Introduction

Approximately 40% of the world’s population provides heating for their homes and cooks their meals using wood-fired heating devices, such as heating or cooking stoves [1]. Considering both wood-fired heating stoves (WFHS) and wood-fired cooking stoves (WFCS), they can be used as heat sources in micro-scale combined heat and power generation systems (mCHP). Such devices can be equipped with thermoelectric generators (TEGs), that directly convert heat into electricity. TEGs have such advantages as direct transfer of heat to electrical energy, lightweight, silent operation, and lack mechanical vibrations [2,3]. On the other hand, they are characterized by relatively low performance (~7%). Such a low performance is related to two main factors: (1) the material’s thermoelectric properties, which affect the efficiency of TEG [4], and (2) temperature gradient, which is related to the sink’s mass flow rate and heat source, properties of flowing fluid, inlet temperature, and the heat exchangers design [5]. Despite the low conversion efficiency, Unfortunately, problems arise with proper selection and the complicated use of TEGs. It is crucial to develop the new construction of TEGs dedicated to market-available stoves or develop a new construction of a combined heat and power unit (CHP) consisting of a furnace and a TEG.

The TEGs are solid-state heat engines made of two semiconductor materials with primary junctions, known as the n-type and p-type. The most basic TEGs are thermocouples, which comprise pairs of p- and n-type TE materials, electrical bounds in series, and thermally bounds in parallel. Electrical conductors, normally copper strips, connect the two legs to one side, creating junctions [6]. Due to construction and operational parameters, the possibility of application of TEGs is really wide and includes such options as low-power appliances, biomedical devices, remote wireless sensors, space power and cooling, consumer applications, microcontrollers, and large-scale waste heat recovery [7]. The TEGs dedicated to working with wood-fired heating stoves should consist of three key elements:

- heat exchanger, which absorbs the heat generated in the stove and transfers it into the thermoelectric (TE) modules,
- TE modules, which generate electricity when there is a temperature difference between their ends,
- heat sink dedicated to dissipating heat from TE modules.

**Figure 1:**
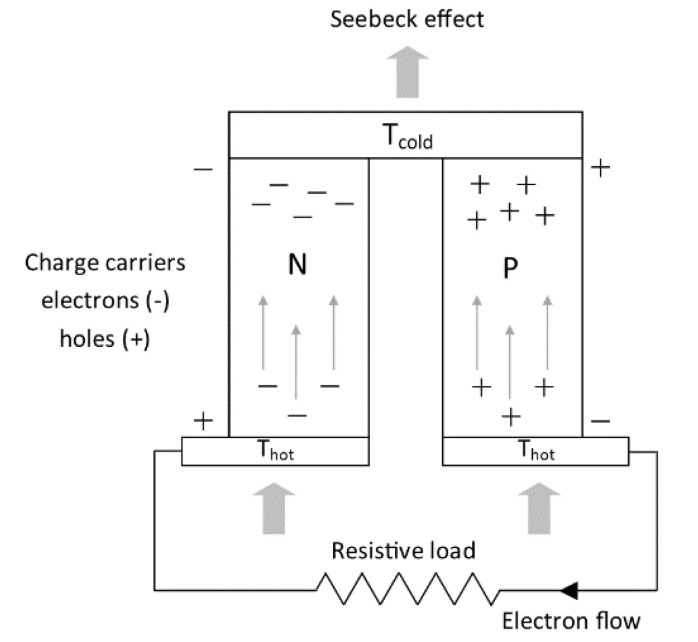
The principle of thermoelectric generation.

The principle of thermoelectric generation is shown in Figure 1.

**Figure 2:**
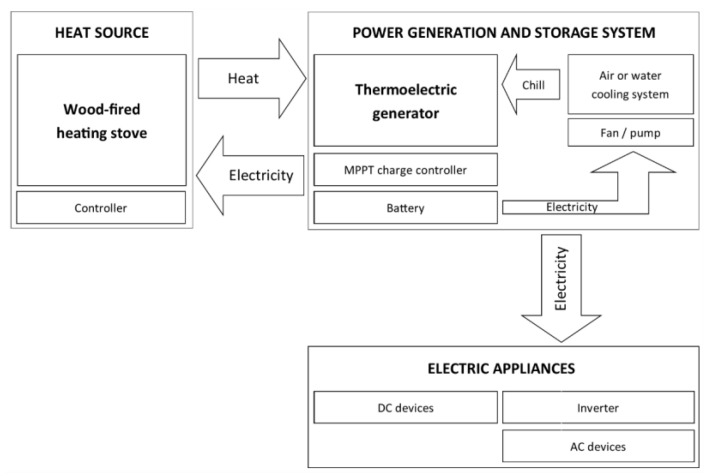
The idea of an mCHP system with a wood-fired heating stove and a TEG.

The proposed system consists of the TEG, the voltage converter (supplying voltage at a 12/24 V DC level at its output), the battery charge controller, the battery, and the controller (combustion optimizer). The generated power is consumed by the controller, air throttle servomotor, and cooling system, which provides a clean combustion process and reduces greenhouse gas emissions (GHG) to meet the targets established at the Paris Conference of Parties 21 (CoP21), and decrease dependence on fossil fuels in EU member countries (EU27) [8]. Surplus power generated in TEG can be used to power the circulation pump in the central heating/hot water system or other home appliances (using an inverter to convert DC to AC power) [9]. The possibility of implementation of TEG in the stove is shown in the scheme presented in Figure 2.

There are some works in the literature that present applications of TE modules to wood-fired heating stoves, cooking stoves, and multifunctional stoves. The example of integration of TEG with the cooking stove was shown by Mal et al. TEG was able to run the small DC fan installed on a chimney. The authors also considered other functions, such as lighting and battery charging [10]. Patowary and Baruah presented the preliminary tests of the TEG-integrated cookstove that showed the potential to generate 2.7 W_e_ of electrical power and illuminate a 3 W_e_ LED bulb [11]. Mastbergen et al. showed a prototype of a TEG cooled by a 1 W_e_ fan [12]. Sornek et al. developed and tested a TEG dedicated to working with the stove-fireplace with accumulation. As was shown, the performance of the TEG was strongly dependent on the cooling method used, and the maximum performance at a level of 5.3 W_e_ was achieved when the water-cooling system was applied [13,14]. Rinalde et al. also studied a forced water-cooling system. During the tests conducted, the maximum power of 10 W_e_ was obtained [15]. Champier et al. studied an mCHP system with a TEG heated by the multi-function stove. The performance of a developed TEG has been established at a level of 9.5 W_e_ [16]. Favarel et al. developed the mCHP system with eight TEMs estimated to produce electrical power at a level of approximately 28 W_e_ [17]. Liu et al. showed that a power generator, comprising 96 TEG modules, had an installed power of 500 W_e_ at a temperature difference of ca. 200 K and an output power of ca. 160 W_e_ at a temperature difference of 80 K [18]. O’Shaughnessy et al. performed an 80-day field test on a cooking stove equipped with air-cooled TEG (fan with an electrical power of 0.5 W_e_) [19]. This TEG provided 3 Wh_e_ a day during the investigations carried out. Guoneng et al. designed and tested the mCHP system based on a TEG that was characterized by a maximum power output larger than 200 W_e_, and heating power larger than 9.8 kW_th_, simultaneously. A strategy interlinking the heat collection, TEG wiring, electricity conditioning and storing, and temperature control, was proposed. Heat collection efficiency for the present system was found to be 34.16%; power generation efficiency was 0.87% and TE efficiency was measured at 2.49% [20]. Another example of a domestic thermoelectric cogeneration system, developed to simultaneously heat water and produce electricity, was shown by Jaber et al. The TEGs were placed on the inner and outer walls of the tank and the pipe. The power produced by TEG located in all layers was at a level of 52 W_e_ and the hot water had a temperature of 81 °C [21]. A high-capacity portable biomass-combustion-powered TEG was developed and tested in [22]. The proposed system was able to cogenerate a heating power of 750 W_th_ and an electric power of 23.4 W_e_, corresponding to a combined heat and power efficiency of 32.3%. Furthermore, it was demonstrated that a 3.7 V battery of 6.2 Ah can be fully charged by burning 1 kg of wood sticks. Najjar and Kseibi presented a heat transfer analysis of a multi-purpose stove coupled with 12 TE modules. This analysis comprised a well-aerodynamically designed combustor, finned TEG base plate, cooker, and water heater beside the outer surface for space heating. It was found that the maximum power obtained is around 7.88 W_e_ use wood, manure, or peat with an average overall efficiency of the stove around 60% [23].

The authors in [24] presented the design and experimental tests of a mesoscale combustor-powered TEG with enhanced heat collection. The electric power was obtained at a level of 25.7 W_e_, while the overall efficiency of 2.69% was calculated. System effectiveness, which was defined as the ratio of overall efficiency to TE efficiency, reached 81.8%. A self-powering and self-aspirating combustion-powered TEG which burns gas fuels was designed and optimized by Li et al. The proposed system was characterized by the possibility of providing simultaneously electric and heating powers of 20.5 W_e_ and 612.6 W_th_, respectively. The overall efficiency of power generation and CHP was determined as 3.0% and 92.5%, respectively [25]. The possibility of using TE modules for power generation in rural Vietnam was analyzed by Anh Do and Mona. A simulated thermoelectric application was carried out using an electronic heater and cooling bath in a modeling conjunction. In the simulation of the external load, the maximum power of TE modules produced up to 331.24 mW_e_ at a gradient temperature of 50 K, whereas in the actual field test up to 63.72 mW_e_ of gradient temperature of about 13.7 K was produced [26]. Another example of the mCHP system—a TEG based on a catalytic combustor—has been proposed in Ref. [27]. Catalytic combustion provides the possibility to profit from the high power densities of hydrocarbon in limited space and low burning temperatures meeting the needs of the. The measured TEG efficiency was achieved at a level of 3.4% with an electrical power output of 5.3 W_e_. In addition to thermoelectric generators, biomass-fired cogeneration systems can operate, for example, according to the organic Rankine cycle or the Rankine cycle. There are also available hybrid systems that integrate different renewable energy sources (such as solar energy, wind energy, water energy, or geothermal energy) [28,29,30].

Apart from experimental work, the same examples of numerical modeling and simulations are also available in the worldwide literature, including analysis based on the usage of CFD tools. CFD tools may be used, e.g., to perform heat transfer and fluid flow simulations to study the thermal characteristics of designs and simulate accurately detailed fluid flow behavior. The use of CFD tools is useful for researchers, designers, engineers, and analysts from the point of view of making decisions earlier in the engineering design process [31,32]. The example of using mathematical modeling to evaluate the behavior of the TEG was presented in [33]. Luoa et al. developed a converging heat exchanger with an attached TEG. The analysis indicated the operational conditions that allow the production capacity of the converging thermoelectric generator device to increase by around 5.9%. Another example of such works was presented in [34]. The three-dimensional governing equations for the flow and heat transfer were solved using CFD methods in conjunction with the thermoelectric characteristics of the TEG over a wide range of flow-inlet velocities. The results showed that at a low flow inlet velocity, the maximum net power output in the TEG with plate-fin heat sink was higher, while the TEG with cross-cut heat sink had a higher maximum net power output at a high flow inlet velocity. Furthermore, Şevik and Özdilli [35] improved the thermal resistance of standard parallel plate heat sinks by developing two types of trapezoidal curved dolphin fins heat sinks and of standard cross-cut parallel plate heat sinks by splaying some of the fins, respectively. Another study devoted to a numerical simulation of the TE module that collects the waste heat from flue gas was presented in [36]. The TEG was modeled as a heat sink that absorbs heat from flue gases in a single and dual system. The results showed that the dual TEG produces 43% more power than a single system, but the overall efficiency is lower. However, this negative impact can be significantly reduced by implementing some changes in the geometry of the channel.

Pujol et al. developed a semi-analytical model to predict the maximum net electrical power of a single TE module with a plate-fin heat sink with non-bypassed forced convection. It was applied to determine the heat sink design that optimized the net electrical power for given values of hot source temperature, TEG properties, and duct cross-section. The optimal heat sink designs predicted by the model for the cases studied had fin thicknesses of 0.32 and 0.44 mm with fin-to-fin distances of 1 mm [37]. Qasim et al. developed a CFD model for a TEG consisting of five TE modules embedded between two aluminum blocks. The model predictions were compared with the authors’ previously published experimental results to assess their validity and reliability. The parametric study revealed a slight inverse relationship between the thickness of the solar-collecting mass, the efficiency of the system, and an increase in the heat flux. However, the relationship was proportional to the velocity of water flow [38]. In [39], the authors examined the effect of aspect ratio and tilt angle on the performance of a TEG located in a chimney. When the number of TEGs was limited or the available surface area is large, the increase in the distance between TEGs to four lengths enhances their power by about 45%. Furthermore, the higher the tilt of the chimney wall towards the flue gases, the greater the output power that is generated. The maximum tilt angle equal to 15° increased the power of the TEGs by around 5%. The effect of the hot-side and cold-side temperature of TEGs on their net power was investigated theoretically and numerically in [40]. The authors stated that the temperature of the critical impact on the TEGs hot side has a tilt angle. The larger the angle, the larger the temperature of the improvement on the hot side. Furthermore, the optimal flue gas mass flow rate should be greater than 37.25 g/s, which increases the net power by 5.96%. Obernberger et al. developed a micro CHP system based on a wood pellet stove (thermal capacity 10.5 kW_th_) with a thermoelectric generator, that enables the operation of the stove without an electric grid connection. To achieve a self-sustaining operation of the stove a TEG with an electric power of up to 50 W_e_ was installed and the electricity consumption has been reduced significantly by an optimization of the control system and the selection of appropriate low voltage components. Based on transient system calculations and CFD simulations as well as test runs with two testing plants the system has been optimized. Overall efficiencies up to 92.6% were achieved and in addition to the coverage of the own electricity consumption of the stove, 50 Wh_e_ surplus electricity was produced during 8 h load cycle tests [41]. Researchers in [42] studied a 50–100 W_e_ TEG fired with natural gas that may be used as an independent and reliable source of power for systems that are not connected to the electric grid. This paper was focused on geometry optimization, which aimed to maximize the temperature difference between the hot and cold sides of the TEGs and maintain the temperature below the required technical limit of 593 K. The authors have chosen the parameters that have the greatest impact on the power output: length of the upper finned zone and exhaust duct, fin thickness, burner thermal output, and deflector width. However, the research included in [43] presents a concept of the heat exchanger that could enhance the cooling effectiveness of the TEGs without the consumption of any electricity. The idea is to apply to utilize the chimney effect to improve heat transfer from the fins. The authors experimentally measured that their solution could increase the power of a TEG up to 46.2% compared to conventional passive cooling systems. The usefulness of CFD methods to increase TEGs performance was discussed in [44]. The authors investigated the impact of the heat exchanger material, inclination, and exit gap of the heat deflector on the generated power. In conclusion, they stated that the TEG has good performance with copper material as heat exchanger material, a 2° inclination angle of the deflector, and a 10 mm exit gap between the heat deflector and the exhaust pipe. The above examples and other available works confirm the validity of further research regarding the use of TEGs in mCHP systems. As was previously proven, the integration of existing stoves and TEGs is very difficult [45]. Therefore, the scope of the proposed work is to expand the knowledge regarding the mCHP systems with TEGs by presenting an experimental and numerical investigation of a prototype installation. The novelty of this work is connected with the proposal of low-price TEG dedicated to operation with a wood-fired stove, that can provide self-sufficient operation of the developed mCHP unit fully or partially network-independent operation of the hot water and heating systems.

## 2. Materials and Methods

### 2.1. Numerical Model

The numerical model was prepared using the commercial software ANSYS 19.1 in a transient state. During the pre-processing, the geometry in the 1:1 scale was prepared in Design Modeler, an ANSYS Workbench module (manufactured by Ansys, Inc., Canonsburg, PA, USA). It represented a steel sheet mounted on the flue gas channel, which collects and stores the heat extracted from exhaust fumes. The cuboidal geometry was discretized in the Mesh module with a maximal mesh element size equal to 0.2 mm. This resulted in a mesh with 61.2 thousand nodes and 50 thousand elements. The mesh quality was stated based on the skewness parameter. Due to the regular shape, the average skewness is nearly equal to 0 and the maximum value is not exceeding the 0.950 limit. The sheet material was set based on the steel properties saved in the Fluent material database. Time-dependent boundary conditions such as fume temperature and ambient temperature were established based on the experimental results. For preliminary numerical analysis, only the first 10 min of stove operation were chosen since the flue gas temperature was lower than 320 °C. As shown in [43], this is a safe temperature limit for the application of TEGs. The measured flue gas temperature was implemented in the solver as a time-dependent user-defined function (UDF) based on polynomial regression. In addition, the local heat transfer coefficient on the cold side of the metal sheet was calculated, similar to the study presented in [38]. The calculation method was based on the dimensionless approach and similarity numbers. To calculate the heat transfer coefficient *α* from the plate with characteristic length L to the air with thermal conductivity *λ*, the Rayleigh number *Ra* should be known. It describes the behavior of fluids when the mass density is non-uniform due to temperature differences. Based on values of the Rayleigh number, the empirical parameters of natural convection (*c* and *w*) may be adjusted:

(1) $$\alpha =\frac{c\lambda \;Ra^{w}\;}{L}$$

The Rayleigh number is calculated by multiplying the values of the Prandtl and Grashoff numbers:

(2) Ra=Pr×Gr

The Prandtl number describes the relationship between momentum diffusivity and thermal diffusivity for air, and it was set as:

(3) $$Pr=\frac{\mu c_{p}}{\lambda }$$

The Grashof number, which is a ratio between the buoyancy forces, and viscous forces were calculated as:

(4) $$Gr=\frac{L^{3}\rho^{2}g\beta \left(T_{a}-T_{o}\right)}{\mu^{2}}$$

To solve the governing equations, the coupled algorithm with the second-order upwind schemes was adopted. This basic model was used to state the thickness of the steel sheet which allows the excess heat when the stove is not working or the combustion process is limited. Moreover, future model development assumes extending the solver functionality by code which allows calculating the power generated by TEG.

### 2.2. Experimental Rig

The experiments were carried out using a test rig equipped with:

- wood-fired heating stove designed for burning seasonal hardwood, characterized by efficiency at a level of 81% and nominal heating capacity at a level of 12 kW_th_ (during normal operation, this power range from 8 to 16 kW_th_);
- prototypical construction of the TEG provided for installation on the flue gas chimney and equipped with four TEMs connected in series and characterized by the characterized by total matched power of 28.8 W_e_;
- dedicated control and measurement system equipped with:
  ○ PLC controller (manufactured by WAGO GmbH & Co. KG, Minden, Germany) with a set of I/O modules;
  ○ temperature sensors provided for monitoring the temperature of flue gas inside the combustion chamber, flue gas at the inlet and outlet from the TEG, and air at the inlet to the combustion chamber; including the K-type (NiCR-Ni) thermocouple sensors with a measuring range from −40 °C to 1200 °C and accuracy ±2.2 °C or ±0.75%, and the Pt100 resistance sensors with a measuring range from −50 °C to 400 °C and tolerance ±0.3 + 0.005 × [t];
  ○ thermoanemometer provided to measure the speed of air blowing to the combustion chamber;
  ○ throttle with servomechanism controlled by analog 0–10 V signal to control the amount of air flowing to the combustion chamber;
  ○ flowmeter provided to measure the water flow in the water-cooling system;
  ○ transducers with measuring range from 0 to 400V DC and measuring error lower than ±0.5% for voltage and with measuring range from 0 to 15A with measuring error lower than ±0.5% for current;
  ○ PC with CoDeSys software.

**Figure 3:**
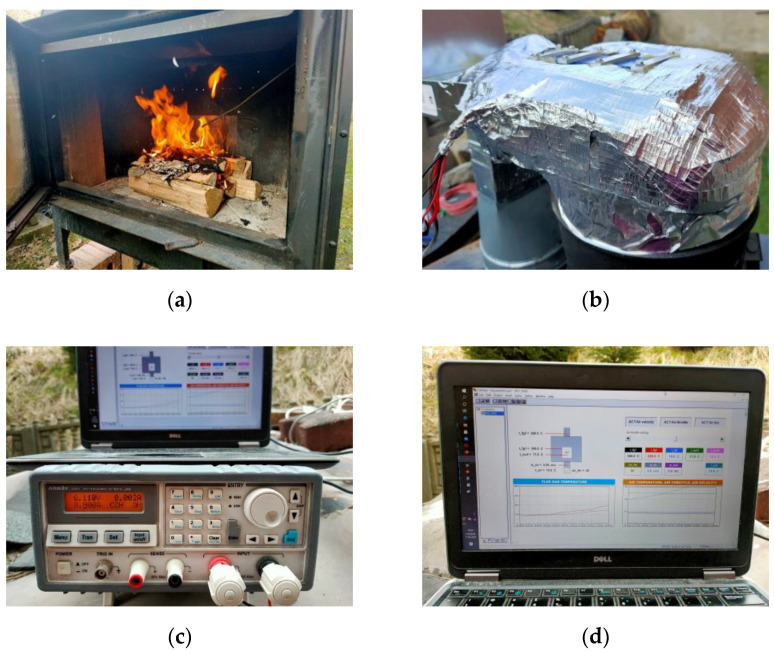
Key elements of the experimental rig: (**a**) wood-fired heating stove, (**b**) TEG prototype, (**c**) electronic load, (**d**) a PC with displayed visualization created in CoDeSys software.

Measurements were recorded using a modular control and measurement system with a PLC controller in a 1s time interval [46]. The PLC controller was also used to set the air throttle (from 0%—fully closed to 100%—fully open). To enable easy control and observation of data collection by the PLC controller, a dedicated visualization was created in CoDeSys 2.3.9.46 software. The key elements of the experimental rig are shown in Figure 3.

During the presented tests, 8 kg of pinewood with a moisture content of 12–13% and a lower heating value (LHV) of approximately 4.4 kWh/kg was burned.

#### The Construction of the TEG Provided for Installation in the Chimney

**Figure 4:**
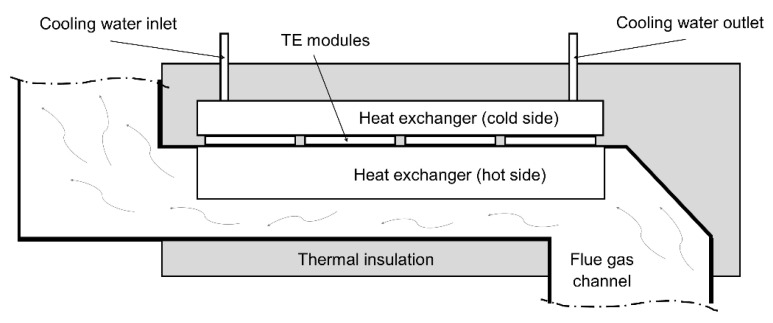
The simplified scheme of the developed TEG.

The hot side of the developed TEG was made in the form of a rectangular channel with a radiator. The shape of the exchanger was developed to increase the heat flux from flue gas to the hot side of TE modules (a change in flow direction by 90° provides better contact with the hot side of the TEG). The radiator was used to increase the surface of the heat transfer from the gases to the exchanger. TE modules were mounted on the radiator using thermal conducting paste. Furthermore, to eliminate excessive cooling of the exchanger surface, thermal insulation was applied, i.e., a 50 mm layer of thick mineral wool. The cold side of the TEG was equipped with an alumina heat exchanger (water was used as the cooling medium). The simplified scheme of the proposed TEG (prototype version) is shown in Figure 4.

**Figure 5:**
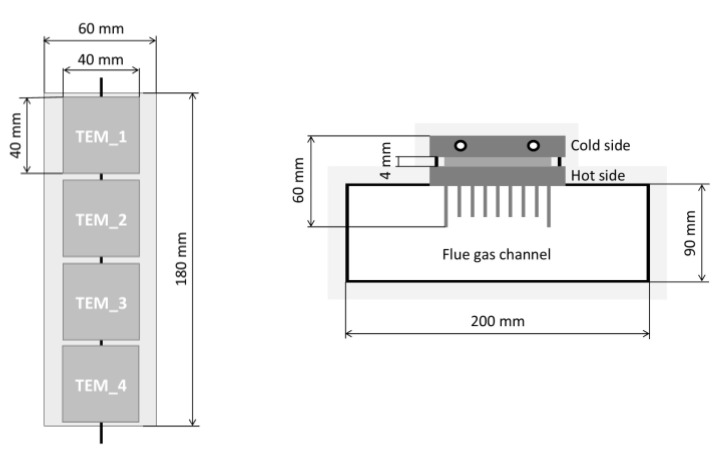
The structure of the developed TEG and its main dimensions.

In the case of the developed thermoelectric generator, a serial connection of TE modules was used. The structure of the developed TEG and its main dimensions is shown in Figure 5.

## 3. Results and Discussion

### 3.1. Numerical Results

**Figure 6:**
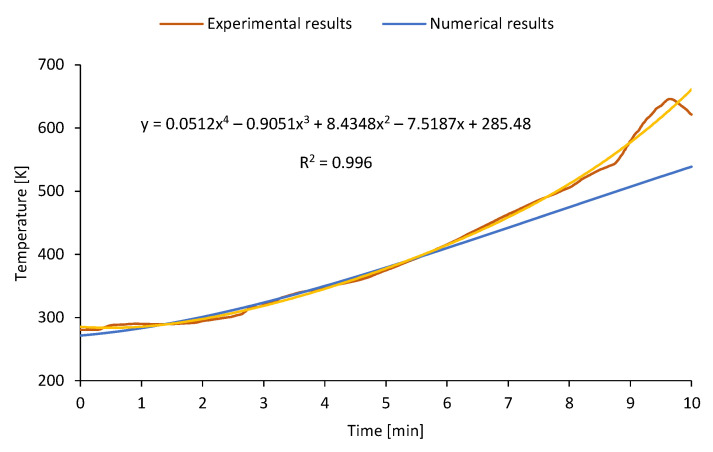
Comparison between the temperature of the flue gas measured during the experiment, the regression curve, and the numerical output.

The temperature functions on the model boundaries were based on the experimental results. Due to the temperature fluctuations that can be observed in Figure 6, the general trend in the temperature of the flue gas was established as a four-degree function with the goodness of fit R2 = 0.996. Then, the polynomial function was coded in the UDF script and implemented in the solver in interpreted mode. It resulted in the divergence between the implemented profile and the solver output.

**Figure 7:**
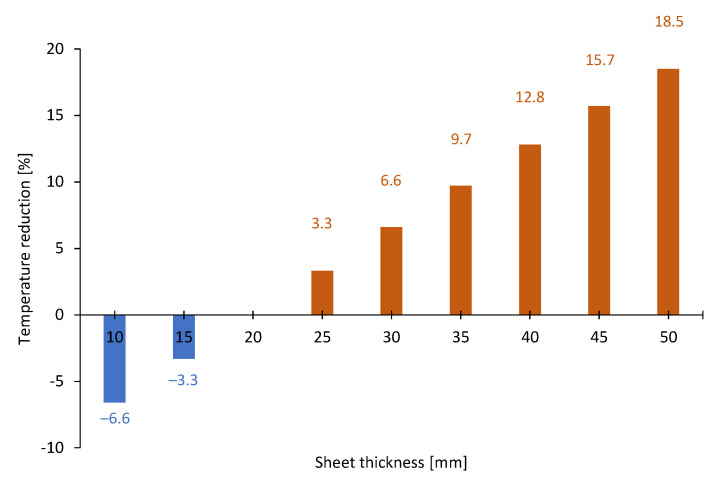
The reduction of temperature on the second side of the metal sheet with the increase in its thickness.

Then, steady-state variant analysis was provided with boundary conditions corresponding to the maximum operating temperature, i.e., 593 K. These simulations were designed to check how the location of the hot side exchanger of the TEG and thickness of the metal sheet influences the temperature of the cold side of the metal sheet (see Figure 7). The proper choice of the thickness of the metal sheet is important both from the standpoint of the level of temperature, which can be achieved, and the thermal inertia. It can be seen that the thicker the sheet, the lower the temperature is. In the basic case (20 mm), the sheet heats up to 593 K, while the usage of the 50 mm-thick sheet reduces this temperature by ca. 18.5% to 483 K. On the contrary, the application of a 10 mm plate results in a higher temperature on the cold wall—632 K. Therefore, the sheet thickness can be used as one of the methods to provide the required temperature of the surface where TE modules are mounted.

### 3.2. Experimental Results

#### 3.2.1. Fluctuations in the Temperature of the Flue Gas and Cooling Water

**Figure 8:**
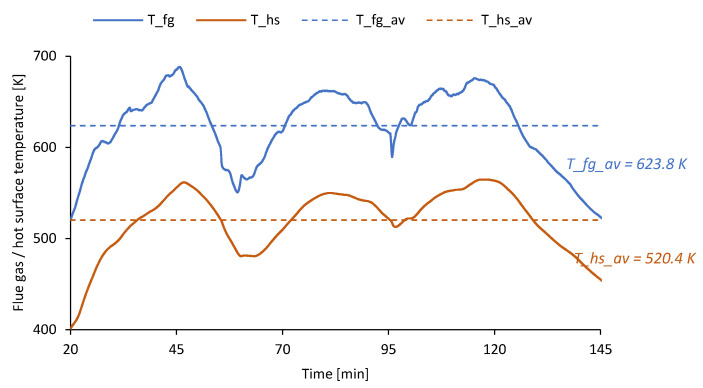
Variations in the temperature of the flue gas (*T_fg*) and hot surface (*T_hs*) during the main phase of the analyzed combustion process.

Voltage, current, and power generated in TEG vary along with the variations in the temperature of the hot and cold sides of the unit. During the main phase of the analyzed combustion process (that is, between minutes 20 and 145), the average temperature of the flue gas (*T_fg_av*) was approximately 623.8 ± 2.6 K, while the maximum temperature (*T_fg_max*) achieved a level of 688.5 ± 3.1 K. Consequently, the average value of the hot surface temperature (*T_hs_av*) was approximately 520.4 ± 1.3 K and its maximum value (*T_hs_max*) was 564.8 ± 1.8 K. The variations in flue gas and hot surface temperature during the main phase of the analyzed combustion process are shown in Figure 8. It can be observed that hot surface temperature directly results from the temperature of the flue gas. On the other hand, the average difference between the temperature of the flue gas and the hot surface is 103.4 ± 2.9 K. It shows, that construction of the hot side of TEG should be optimized to increase the heat flux from flue gas to TE modules. Furthermore, to increase the efficiency of TEG operation, the fluctuations in the flue gas temperature should be limited by implementing better control of the stove operation (both from the standpoint of fuel feeding and control of an air throttle opening).

**Figure 9:**
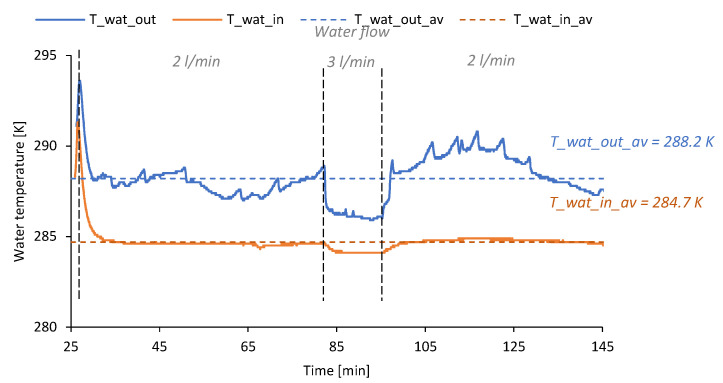
Variations in the temperature of water at the outlet from the TEG (*T_wat_out*) and water at the inlet to the TEG (*T_wat_in*).

When analyzing the variations in cooling water temperature it can be observed that the temperature measured at the outlet from TEG (*T_wat_out*) during the main phase of the analyzed combustion process varied from 285.9 ± 0.4 K to 293.6 ± 0.4 K, and the average value of the water at the outlet from the TEG (*T_wat_out_av*) was approximately 288.2 ± 0.4 K. On the other hand, the average difference between the temperature of water at the outlet and the inlet to the TEG was approximately 3.5 ± 0.3 K. The variations in the temperature of water at the outlet from the TEG (*T_wat_out*) and water at the inlet to the TEG (*T_wat_in*) are shown in Figure 9. Considering the water flow ranged from 2.0 to 3.0 L/min, the heat transferred to the water during the main phase of the stove operation was approximately 1 078.0 ±182.2 Wh_th_.

#### 3.2.2. Operating Characteristics of the Developed TEG

**Figure 10:**
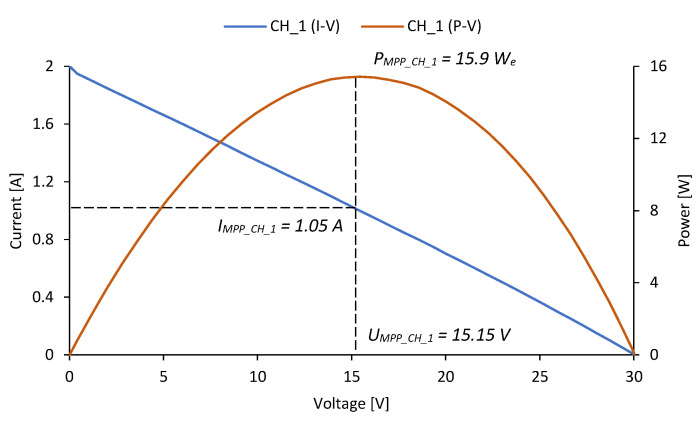
I–V and P–V characteristics of the tested TEG when the flue gas temperature was 620 K.

The current–voltage and power–voltage characteristics of the developed TEG have been determined during the analyzed combustion process for various temperatures of the flue gas. Characteristics presented in Figure 10 have been determined for temperature 620 K corresponding to the average flue gas temperature (series *CH_1*). As can be observed in Figure 10, the short circuit current (*I_sc_CH_1_*) achieved a maximum value of 2.0 A, and the open circuit voltage (*U_oc_CH_1_*) achieved a maximum value of 30.1 V. As a result of the measured current and voltage values, the P–V characteristics were determined. The maximum power attained (*P_MPP_CH_1_*) was 15.9 ±0.1 W_e_ for a matched load current (*I_MPP_CH_1_*) of 1.05 A and a matched load voltage (*U_MPP_CH_1_*) of 15.15 V.

The expected level of energy generated in the developed TEG during the considered combustion process can be estimated at a level of approximately 33.1 ± 0.2 Wh_e_. It is possible to reach by providing proper hot and cold sides temperature as well as MPPT system operation. However, in the case of partial wood load, flue gas temperature can be lower, which will limit the amount of generated power. Thus, the impact of the flue gas temperature on the generated electric power has been tested.

#### 3.2.3. The Impact of the Temperature of the Flue Gas on the Generated Electric Power

**Figure 11:**
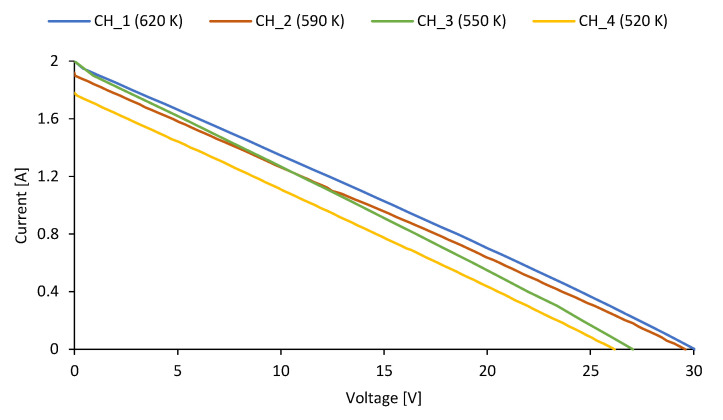

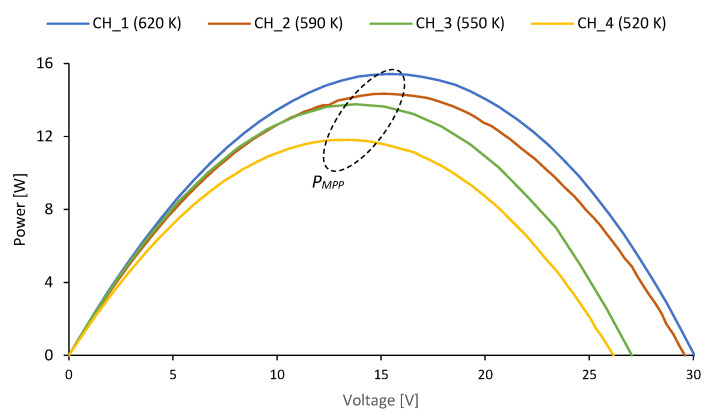
I–V and P–V characteristics of the tested thermoelectric generator.

The I–V and P–V characteristics for different temperatures of the flue gas (*T_fg*), including 620 K, 590 K, 550 K, and 520 K, respectively, are shown in Figure 11. Compared with the series *CH_1*, the generated power was lower by 8.2%, 14.5%, and 25.8% when the flue gas temperature was 590 K (series *CH_2*), 550 K (series *CH_3*), and 520 K (series *CH_4*), respectively.

Table 1 presents the essential parameters of TEG depending on the flue gas temperature, including open circuit voltage, short circuit current, matched power, matched voltage, and matched current.

**Figure 12:**
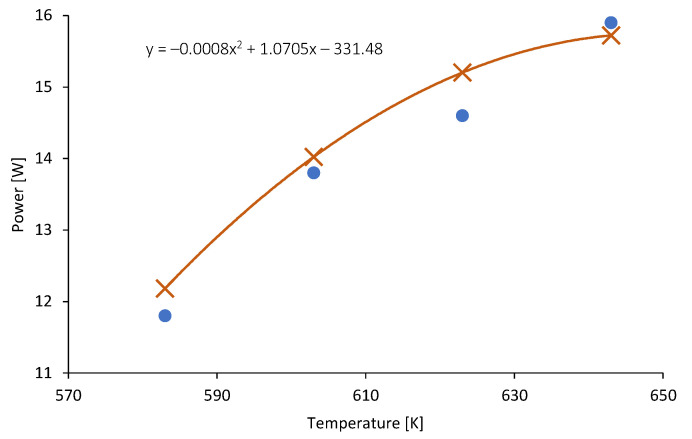
Variations in the generated power as a function of flue gas temperature.

As can be observed in Figure 12, the dependence between the flue gas and the power generated in TEG can be estimated by a polynomial function with very good precision (R2 = 0.995). The proposed function can be used to calculate the level of power generated in the TEG depending on the flue gas temperature (in the typical operating conditions of the analyzed wood-fired stove).

#### 3.2.4. Energy and Economic Aspects of the Integration of TEG with a Stove

During the typical operation of the analyzed wood-fired heating stove, the controller takes a power range from 1.5 to 5.0 W_e_. Moreover, additional power is necessary to drive the water circulation pump. Taking into account the duration of the main phase in the analyzed combustion process, the power consumption can be assumed at a level of approximately 20.8 Wh_e_, while the available electric generation is approximately 33.1 Wh_e_. It can be stated that the current version of the TEG can provide the self-sufficient operation of the tested stove. With the addition of more TE modules, the generated power can be stored in the battery for further usage.

Assuming two options: the introduction of an actual version of the TEG (case A) and the introduction of a modified TEG with 16 TE modules (case B), further analyses were conducted. By increasing the number of TE modules, the efficiency of the power generation can be improved with the simultaneous reduction of chimney losses. The available electric generation will increase to approximately 130 Wh_e_, when a single combustion process is considered. However, this value will be still only a really small amount compared to the chemical energy included in the wood. Thus, the proposed system can be considered only as an additional power source allowing for fully or partially network-independent operation of the hot water and heating systems.

The essential parameters of the TEGs assumed by the economic analysis are shown in Table 2.

In the case analyzed, the operational costs of the proposed mCHP system are related to fuel costs and the cost of energy consumed by the controller, as well as additional costs of energy consumed by the TEG water-cooling system. The actual price of pinewood in Poland is approximately 20 ÷ 32 EUR per stack m^3^, while the actual price of electricity was assumed to be at a level of 0.2134 EUR per kWh_e_ (based on the average price in the EU-27 area for the second half of 2020). Taking into account the above-mentioned data, the economic indicator in the form of Simple Payback Time (SPBT) was calculated. A building with an area of 150 m^2^ and a heat demand of 70 kWh_th_/(m^2^a) was adopted as the reference building. The main heat source is a natural gas-fired boiler and the building was equipped with a wood-fired heating stove as an additional heat source. Considering an installation of TEG to the existing stove, the SPBT will be, respectively, equal to approximately 28.9 years (in case B) and 66.1 years (in case A). However, the SPBT will be significantly shorter, when the installation of the whole mCHP system will be considered as an additional heat and power source (approximately 5.4 years in case B). Taking into account the above calculations, the installation of the TEG is an expensive option. However, it should be noted that the introduction of the mCHP system can provide fully or partially network-independent operation of the hot water and heating systems. It is important to take into account the increasing prices of fossil fuels and electricity, as well as from the standpoint of avoiding possible periodic interruptions in the access to heat and electricity from the public grid. On the other hand, further development of TEGs parameters should increase their performance and lower investment costs.

## 4. Conclusions

Power generation using TEG is increasing in popularity due to the decrease in the prices of TE modules and the high costs of consuming electricity from the grid. This paper includes the results of numerical and experimental investigations. The novelty of this work was connected with the proposal of low-price TEG dedicated to operation with a wood-fired stove, that can provide self-sufficient operation of the developed mCHP unit fully or partially network-independent operation of the hot water and heating systems. The main findings resulting from the conducted works are as follows:

- Mathematical modeling made it possible to analyze different configurations of the hot side of the TEG. Simulations were designed to check how the location of the hot side exchanger of the TEG and the thickness of the metal sheet influences the temperature of the cold side of the metal sheet.
- The maximum power obtained during the discussed combustion process was observed at a level of 15.9 W_e_ when the flue gas temperature was approximately 623 K.
- The energy generated in the TEG during the considered combustion process was estimated at a level of 33.1 Wh_e_, while the heat transferred to the water was calculated as approximately 1 078.0 Wh_th_.
- Compared to the maximum observed power, the generated power was lower by 8.2%, 14.5%, and 25.8% when the flue gas temperature was 590 K, 550 K, and 520 K, respectively.
- It was stated that the current version of the TEG can provide the self-sufficient operation of the tested stove. With the addition of more TE modules, the generated power can be stored in the battery for further usage.
- Assuming two options: an introduction to an actual version of the TEG and an introduction to modified TEG with 16 TE modules, an economic analysis was conducted. Considering the installation of TEG to the existing stove, the SPBT will be about 28.9–66.1 years. On the other hand, the SPBT would be significantly shorter, when the installation of the whole mCHP system was considered (approximately 5.4 years).

Summarizing the results obtained, further investigation should be focused on providing a stable temperature of the hot side of the TEG. It can be realized by developing a dedicated controller, which will control the stream of air blown to the combustion chamber. Furthermore, TE modules characterized by higher efficiency and higher number should be introduced in the subsequent version of the TEG. After these modifications, field tests should be performed to assess the real performance and efficiency of the heating and electricity generation during operation in real conditions.

## Figures and Tables

**Table 1:** The essential parameters of TEG depending on the flue gas temperature.

TEG Parameter	CH_1	CH_2	CH_3	CH_4
Flue gas temperature, K	620	590	550	520
Open circuit voltage, V	30.1	29.6	27.1	26.2
Short circuit current, A	2.0	1.9	1.9	1.8
Matched power, W_e_	15.9	14.6	13.8	11.8
Matched voltage, V	15.15	14.17	13.66	13.43
Matched current, A	1.05	1.03	1.01	0.88

**Table 2:** The essential parameters of thermoelectric generators assumed by economic analysis.

TEG Parameter	Case A	Case B
Type of TEMs, -	Low-cost	Low-cost
The number of TEMs, -	4	16
The matched power, W_e_	28.8	115.2
The cooling medium, -	Liquid (water)	Liquid (water)
Estimated cost, EUR	200	350

## Data Availability

Not applicable.
